# Restoring the common hamster’s farmland habitat—how crop associations might benefit *Cricetus cricetus* hibernation and reproduction

**DOI:** 10.1098/rsos.250499

**Published:** 2025-08-27

**Authors:** Timothée Gérard, Hugo Chignec, Aurélie Saussais, Chantal Poteaux, Emilie Long, Jean-Patrice Robin, Sandrine Zahn, Caroline Habold

**Affiliations:** ^1^ Institut Pluridisciplinaire Hubert Curien, Unité Mixte de Recherche 7178, Université de Strasbourg, Centre National de la Recherche Scientifique, Strasbourg 67200, France; ^2^ Office de Génie Ecologique - O.G.E., Strasbourg 67200, France; ^3^ Laboratoire de Biométrie et Biologie Évolutive, Unité Mixte de Recherche 5558, CNRS, Lyon 69622, France; ^4^ Laboratoire d'Ethologie Expérimentale et Comparée, UE 4443, Université Sorbonne Paris Nord, Villetaneuse 93430, France

**Keywords:** common hamster, agroecology, conservation, hibernation, reproduction, agriculture, farmland biodiversity

## Abstract

Biodiversity decline is particularly pronounced in agricultural areas, where intensive farming practices have severely altered the ecosystems. In Alsace (France), this has led to the decline of the common hamster (*Cricetus cricetus*), a farmland-inhabiting hibernator. Previous work in laboratory conditions showed that diversifying the hamsters’ diet through crop association is a promising strategy to improve their hibernation and reproductive success. However, little is known about the effect of such crop associations in the wild. In this study, we monitored the hibernation behaviour (in laboratory cages) and reproductive success (in mesocosms) of hamsters exposed to four different crop associations of variable nutritional content, selected for their technical and economic benefits for farmers. Hamster hibernation behaviour depended mainly on the ingested energy and only marginally on the nutritional quality of the diet. Hamsters on lipid-rich diets showed a higher body mass before reproduction. All hamsters successfully reproduced in semi-natural conditions, even in wheat monoculture, where food supplements (weeds, invertebrates) prevented protein deficiencies. Associations richer in proteins and lipids such as legume-oleaginous crop mixes doubled reproductive outputs and increased pup growth. These results should help to improve hamster conservation measures and promote farmland biodiversity.

## 1. Introduction

Farmland ecosystems are threatened by modern agricultural practices, which are characterized by a production-oriented intensive farming of few crop varieties, cultivated in large monocultural plots [1]. Such practices undermine ecosystem services such as nutrient and water cycling or the regulation of pests [1,2]. This leaves current agricultural practices highly dependent on watering systems and the use of fertilizers and pesticides [3]. In the face of global change and the biodiversity crisis, these practices are thus unsustainable [3,4].

Those aspects are linked to a decline in farmland biodiversity, currently observed from soil microbial communities to weeds, invertebrates, bird and mammal species [5–8]. In Europe, intensive agricultural practices are threatening an emblematic rodent species: the common hamster *Cricetus cricetus* [9–11]. Throughout the twentieth century, drastic population decline was notably driven by pest control policies and the intensification of agricultural practices, causing local populations to almost disappear [12]. Today, the common hamster is listed as ‘critically endangered’ throughout its range [13]. In Alsace (northeastern France), the species has been protected since 1993. Conservation measures for this species are notably based on habitat restoration through the incentive of ‘favourable crops’ (i.e. crops suited to fulfil hamster’s nutritional needs; [14]). Thus, further investigation into hamster’s nutritional requirements and crops adequacy is needed.

Hamsters have a complex annual life cycle, that is divided between an *active season* from spring to fall, and an *inactive winter season* spent inside a burrow [15,16]. In winter, hamsters hibernate, by engaging in sequential torpor bouts (a period we refer to as *‘hibernation’* in this manuscript). Torpor bouts are characterized by decreased metabolic rates and body temperature [15]. They are associated with significant energy savings [17], but also homeostatic challenges, such as immune depression or oxidative damage [18]. Torpor bouts are separated by arousal phases, during which hamsters return to euthermia and feed on stored food, following a *food-storing* hibernation strategy. Indeed, in late summer and autumn, common hamsters hoard large quantities of non-perishable grain and/or tuber in their burrow, that they consume during winter [19] and, if some stored food remains, at the beginning of the reproductive season in spring [16]. Hamsters’ hibernation is also strongly affected by their stored food stocks quality. A high lipid content in the diet has been shown to reduce torpor use and overall hibernation duration, while body condition at the end of hibernation is improved [20,21]. At the end of hibernation, before hamsters will leave their burrow, they undergo a torpor-less period, required to regenerate their reproductive system (a period we refer to as *‘post-hibernation’* in this manuscript; [22,23]). The body condition of hamsters at the end of the winter strongly depends on the food consumption and torpor behaviour during the hibernation period. Body condition of wild hamsters after winter has been declining throughout the last decades [11]. This might be critical, as body condition at this time impacts reproductive success in the following season [20].

The active season of hamsters runs from April to October. During that time, hamsters are active above ground and engage in a number of behaviours, such as prospecting for a partner, foraging, etc. [24]. Until 1950, female hamsters produced up to 3 litters of 8 to 12 pups per year, compensating for the high mortality rate caused by winter conditions and predation [9,25]. In contrast, their current reproductive success is greatly reduced throughout Europe (for example, 0.8 litter of 1 to 3 pups per year in Belgium [26]), including France (0.9 litter of 2.7 pups per year; [27]) and might explain the absence of population recovery. One of the factors contributing to this low reproductive rate is the nutritional deficiencies in the hamsters’ diet. Maize and wheat monocultures, the two main crops cultivated in Alsace, are insufficient by themselves to fulfil females’ protein requirements during gestation [28]. Maize also induces a deficiency in vitamin B3 (niacin) and its precursor (tryptophan), causing infanticide of 95% of the pups by females [10,28]. The susceptibility of hamsters to nutritional deficiencies can be explained by the small home range size of females (from 0.3 to 1.6 ha, depending on habitat quality), restricting them to a monotonous diet in considerably larger monocultural fields [26,29].

Recent studies have identified crop associations as promising to diversify hamster habitat and promote population growth [10]. As example, captive-bred hamsters fed a wheat and soybean diet show a significantly higher reproductive success than those fed with wheat only [20,30]. However, these studies were conducted under laboratory conditions, thus neglecting the importance of environmental factors. Hamsters are omnivorous and can diversify their diet by eating crops’ green parts, weeds and small animals, if available in their environment [10,31]. To date, the only crop association tested in semi-natural conditions was a mix of wheat, maize, sunflower and alfalfa, which is of no commercial interest to the farmers [10]. Hence, it is now crucial to evaluate the effects of economically viable crop associations in conditions that are more representative of the hamster’s wild habitat.

In this study, we investigated the effects of different crop associations on hamster traits, focusing on females (males monitoring is presented in electronic supplementary material). We monitored hamsters (i) during a winter season under controlled laboratory conditions and (ii) during reproduction in a semi-natural environment (mesocosm). The crop associations tested were chosen based on their suitability in the context of habitat restoration measures, and considering their technical feasibility and economic interests for the farmers. We tested two crop associations rich in both lipids and proteins: *WS,* a mix of wheat (*Triticum* sp.) and soybean (*Glycine max*), and *RF*, a mix of rapeseed (*Brassica napus*) and fava bean (*Vicia faba)*. We compared them with two lipid-poor diets with intermediary amount of proteins: *ML*, a mix of maize (*Zea mays*) and lablab bean (*Lablab purpureus*) and *W,* a monocultural wheat diet used as a control treatment. We expected that (i) during winter, lipid-rich diets (*WS* & *RF*) would affect hibernation behaviour, leading to a reduction in torpor use and an earlier emergence from hibernation; (ii) females from these two groups would have higher energy and lipid intakes and, consequently, gain mass during hibernation, leading them to emerge in a better body condition; (iii) the better body condition of these females would benefit reproduction: they would be able to reproduce earlier and produce more pups; (iv) the *W* diet would lead to protein deficiencies, thus preventing the successful weaning of pups; and (v) by contrast, protein-rich diets would increase reproductive output and pups’ growth.

## 2. Material and methods

### 2.1. General set-up

This study was conducted on 32 females and 12 males of common hamsters from our breeding unit and of known pedigree. At the beginning of the monitoring, hamsters were four to seven months old and nulliparous. Hamsters were divided into four groups, consisting of eight females and three males each, with similar mean body mass and standard deviations between groups. They were assigned to one of the four diets: *RF* (rapeseed-fava bean), *WS* (wheat-soybean), *ML* (maize-lablab bean) or *W* (monoculturous wheat; see electronic supplementary material, table S1 for details of the nutritional content of crops). Hamsters were kept under laboratory conditions during winter (period set between 19 October 2021 and 19 April 2022) so that food consumption could be measured. Thereafter, they were released into outdoor enclosures, where reproduction was monitored during the active season (19 April 2022 to 19 October 2022).

### 2.2. Body temperature monitoring

All 44 hamsters were implanted with intraperitoneal iButton temperature loggers (ref. DS1922L, Maxim Integrated), that recorded temperature every 135 min. at a 0.06°C resolution. Surgical procedures followed the protocol described by Weitten *et al.* [21]. Implantation surgeries were conducted three weeks prior to the start of the monitoring period, to ensure the complete recovery of hamsters. Loggers were retrieved following the same surgical procedure after the end of experiment. iButtons could not be recovered from six females that disappeared after initial release (i.e. never recaptured; 1 *WS,* 1 *ML,* 1 *W*) or after reproduction (i.e. not recaptured in September; 2 *W*, 1 *ML*; for details, see electronic supplementary material, table S4).

### 2.3. Hamster housing during hibernation

Hamsters were housed in individual cages (*W* × *L* × *H*: 380 × 590 × 257 mm), and provided with a PVC tube for shelter (⌀ × *L*: 17 × 200 mm, placed vertically and pierced to enable hamster access). Cages were enriched with cellulose sheets for nest building and wooden sticks to gnaw on. Artificial light exposure followed the natural variation of photoperiod at the latitude of Strasbourg, France (48.58° N). Temperature was maintained at 10°C throughout hibernation (corresponding to wild-like conditions, [32]). Each hamster had ad libitum access to its diet. Food consumption was quantified following Gérard *et al*. [30] and outlined in the electronic supplementary material. Daily visual monitoring allowed to distinguish hibernation and post-hibernation periods (see electronic supplementary material, table S2).

Body temperature patterns were used to characterize hibernation. To exclude short and shallow torpor bouts that hamsters may engage in during the active season, torpor was defined as a period during which body temperature dropped below 30°C for a minimum of 24 h [19]. Isolated torpor bouts that occurred more than 15 days after the end of the main hibernation period were not considered in our analysis. The time females spent in torpor during winter correlated with their overall hibernation duration (i.e*.* time between first and last torpor bout, *R*^2^ = 0.725, *p* < 0.001), and the number of torpor bouts (*R*^2^ = 0.934, *p* < 0.001). Accordingly, we used the total time spent in torpor to characterize hibernation behaviour in our analysis.

### 2.4. Mesocosm monitoring

The enclosure used for the study was located near Ittenheim, France (48°36′ N; 7°37′ E). It was divided into four sub-enclosures, with an area of 0.19 ha each. The overall structure was protected by a retaining wall of Larssen sheet piles buried 2 m deep and protruding 1 m above ground. The iron wall was mounted with a fence supporting a net, thus preventing entry of any terrestrial or aerial predators. Sub-enclosures were separated by a 0.4 m high and 2.0 m deep concrete wall. Due to delays in enclosure construction, winter crops could not be sown before winter. Hence, the sub-enclosures were sown with spring growing varieties, mimicking the cover provided by the tested crops, namely spring wheat for the *W* group, spring wheat and soy for the *WS* group and maize and lablab beans for the *ML* group. The *RF* sub-enclosure was sown with winter rapeseed (no spring varieties were available), and peas as a spring replacement for fava beans. Sowing occurred after a superficial 10 cm deep ploughing and seeds were covered by harrowing. Sowing took place on 30 March 2022 and 3 May 2023, following the standard agricultural schedule.

Hamsters were released into the enclosure on 19 April 2022. Crops in each enclosure corresponded to their hibernation diet. To ensure sufficient food availability, 3 l of diet seeds were randomly dispersed in the sub-enclosure every week, throughout the monitoring period, similarly to what was done in previous mesocosm studies [10]. From April to October, trapping sessions were conducted once or twice a week (depending on pup density), so that pups and adults could be identified and weighed. Trapping effort was the same for all sub-enclosures. When trapped for the first time, pups were sedated using isoflurane (1.5% in 1.2 l min^−1^ air) for weighing, radio frequency identification (RFID) microchip injection and hair sample collection [33]. This procedure lasted a maximum of 5 min. Pup awakening was monitored before they were released at the capture site. During each session, trapped adults and already-tagged pups were RFID identified and weighed (to the nearest 0.1 g). Adult mean body mass throughout reproduction was obtained by averaging their mass recorded during all captures of that period (mean: 14 ± 1 points per hamsters). To avoid any potential bias associated with population density or pup reproduction attempts, pups were brought to the laboratory breeding unit when weighing more than 140 g. Adults were taken back to the laboratory breeding unit from 20 July 2022 onward for males and from 01 September 2022 onward for females.

Pup capture was only possible when weaned pups freely exited the burrow. However, an unknown proportion of pups might have died before capture was possible, as post-natal mortality is commonly reported in this species [20,30]. Hence, in the following *‘pups’* refers to the number of trapped pups, not accounting for pups that might have died before weaning. Similarly, *litter size* refers to the number of weaned and trapped pups.

### 2.5. Quantifying reproductive success

Genetic analyses conducted on hair samples allowed the parents of each pup to be identified with a confidence interval of 97% (see electronic supplementary material). Body temperature was used to determine parturition dates, by identifying sudden increase in mean daily body temperature by 1°C. This method has been validated on arctic ground squirrels [34] and on the common hamster in our laboratory [33]. A sequential *litter number* was then assigned to each parturition (i.e. first, second, third or fourth litter). Pups were assigned to a specific parturition date. To do so, pups that were undoubtedly from the first litter (pups captured too early to be from the second litter; 108 out of 320 individuals) were used to estimate standard growth rates (sex and group dependent, in gday^−1^). These standard growth rates were then used to assign pups from later litters to the most likely parturition date. This method appears robust, as inferred birth dates (i.e. computed from growth rates) and actual birth dates (i.e. deduced from female body temperature) only differed by 5.0 ± 0.3 days, while the mean interval between two parturition dates was 30.0 ± 1.3 days. In some cases, a litter was produced but no captured pups were attributed to the parturition, indicating pup mortality. In this case, the litter was counted, albeit with zero pups. For details concerning the reproductive output of individuals, see electronic supplementary material, table S4.

### 2.6. Statistical analyses

All standard variances were computed as standard error of the mean (s.e.m.). Statistical analyses were conducted in R (version 4.3.1; [35]). The significance threshold was set to 0.05. Parametric analyses were preferred if the required conditions were met. Standard linear models (lm) were employed for qualitative or quantitative analyses. For count data (e.g. number of pups), generalized linear models (glm) based on Poisson distribution were used. To account for random effects (e.g. to test body mass effects on reproductive success independently of diet), linear mixed-effects models (lmem or glmem for Poisson distribution) from the package lme4 (version 1.1-23; [36]) were used. Corrected Akaike information criterion (AICc)-based model selection was used to determine relevant effects and identify collinearity between factors. This was done using the dredge function from the MuMIn package (version 1.47.5; [37]). If parametric models were inappropriate, non-parametric qualitative Kruskal–Wallis (KW) or quantitative Mann–Whitney (MW) approaches were used. Post hoc tests were performed using either Tukey or Dunn methods, as appropriate. Graphical representations were computed using the ggplot2 package (3.5.0; [38]).

## 3. Results

### 3.1. Hibernation

#### 3.1.1. Food consumption during hibernation

**Figure 1.**
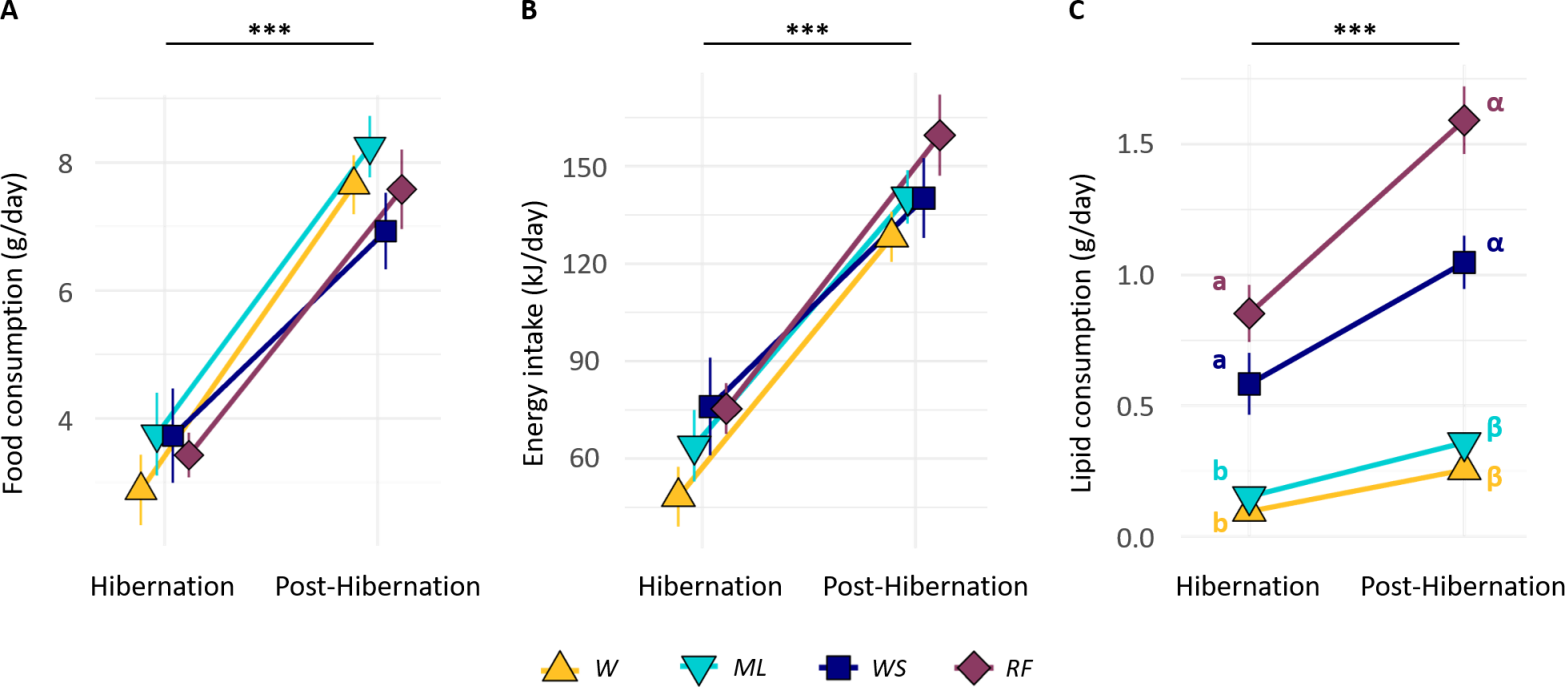
Food consumption (A), energy intake (B) and lipid intake (C) of female hamsters (*n* = 32) according to experimental group and contrasting the hibernation and post-hibernation periods. The different groups are indicated: wheat (*W*), wheat-soybean (*WS*), rapeseed-fava bean (*RF*) and maize-lablab bean (*ML*). The asterisks above indicate statistically significant differences between periods (lm, *p* < 0.001). Values are means ± s.e.m. Latin and Greek letters in (C) correspond to independent statistical analysis comparing groups in each period (Tukey, *p* < 0.02).

During the laboratory winter monitoring, the mean food intake of females across experimental groups was significantly lower during hibernation (3.45 ± 0.89 g day^−1^) than during post-hibernation (7.60 ± 0.27 g day^−1^; lm, *p* < 0.001; figure 1A), and the energy intake varied accordingly (figure 1B; lm, *p* < 0.001). However, intakes did not differ between groups neither during hibernation (KW *food, H* = 1.622, d.f. = 3, *p* = 0.654; *energy, H* = 4.489, d.f. = 3, *p* = 0.180) nor during post-hibernation (KW *food, H* = 3.469, d.f. = 3, *p* = 0.325; *energy, H* = 3.776, d.f. = 3, *p* = 0.287).

**Figure 2.**
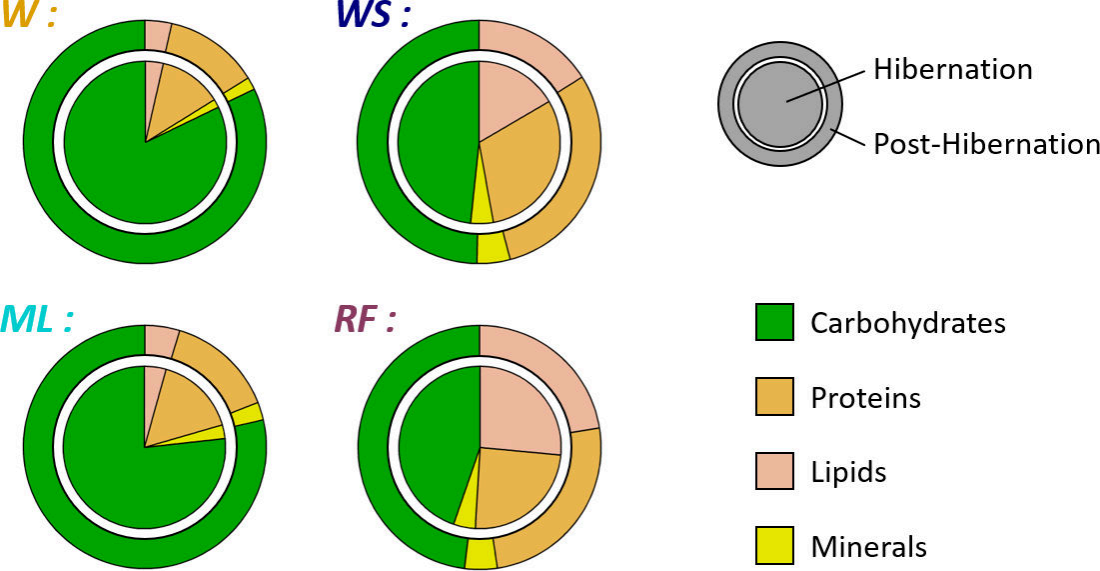
Mean nutritional composition of female hamsters’ intake (dry mass; *n* = 32) during hibernation (inner circle) and post-hibernation (outer circle). Different nutrients are shown by different colours, as indicated in the legend. Details on group differences can be found in the electronic supplementary material, table S2.

*WS* females showed a significant preference for soybeans that constituted 65.1 ± 9.1% of their food intake (MW, *U* = 32, *p* < 0.001). *ML* females preferentially consumed maize that accounted for 65.1 ± 18.6% of their food intake (MW, *U* = 192, *p* = 0.011). By contrast, *RF* females showed no preference (50.8 ± 12.7% of rapeseed, MW, *U* = 96, *p* = 0.205). The food preferences of females and, hence, the composition of their food intake did not differ between hibernation and post-hibernation (MW, *U*_RF_ = 44, *p*_RF_ = 0.235, *U*_ML_ = 27, *p*_ML_ = 0.645, *U*_WS_ = 22, *p*_WS_ = 0.328, figure 2). Macronutrient intake during hibernation and post-hibernation were similar for the *W* and *ML* groups (Dunn *lipids*, *p*_Hib_ = 0.175, *p*_Post-Hib_ 0.110, *proteins, p*_Hib_ = 0.082, *p*_Post-Hib_ = 0.126; *carbohydrates*, *p*_Hib_ = 0.204, *p*_Post-Hib_ = 0.405) and for the *RF* and *WS* groups (Dunn *lipids*, *p*_Hib_ = 0.236, *p*_Post-Hib_ = 0.110, *proteins, p*_Hib_ = 0.228, *p*_Post-Hib_ = 0.426, *carbohydrate, p*_Hib_ = 0.325, *p*_Post-Hib_ = 0.426). However, *WS* and *RF* groups consumed more lipids (3- to 8-fold; figure 1C) and proteins (double) than the *ML* and *W* groups. This difference was significant (Dunn, *p* < 0.02 for both lipids and proteins) except between *RF* and *ML* groups during hibernation (Dunn, *p* = 0.083). By contrast, the carbohydrate consumption of *W* and *ML* groups was double that of the *WS* and *RF* groups during post-hibernation (KW, *H* = 22.287, d.f. = 3, *p* = 0.085; Dunn, *p* < 0.001).

#### 3.1.2. Hibernation behaviour

**Figure 3.**
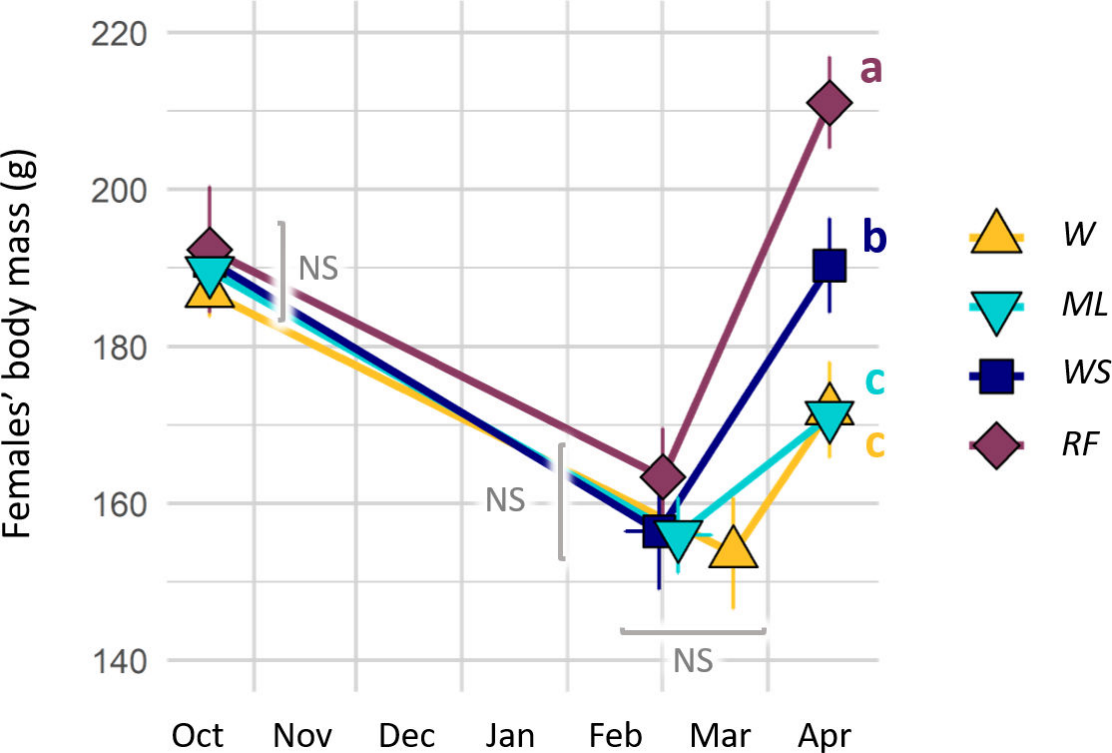
Body mass (g) of female hamsters before (19 October 2021) and after hibernation (second set of points) and at the end of winter (19 April 2022), according to experimental groups. Values are means ± s.e.m. given for each diet group (*n* = 32, with the exception of end of hibernation, where *n* = 31). Letters indicate statistically significant mass differences between groups at each time point (Tukey, *p* < 0.013).

**Figure 4.**
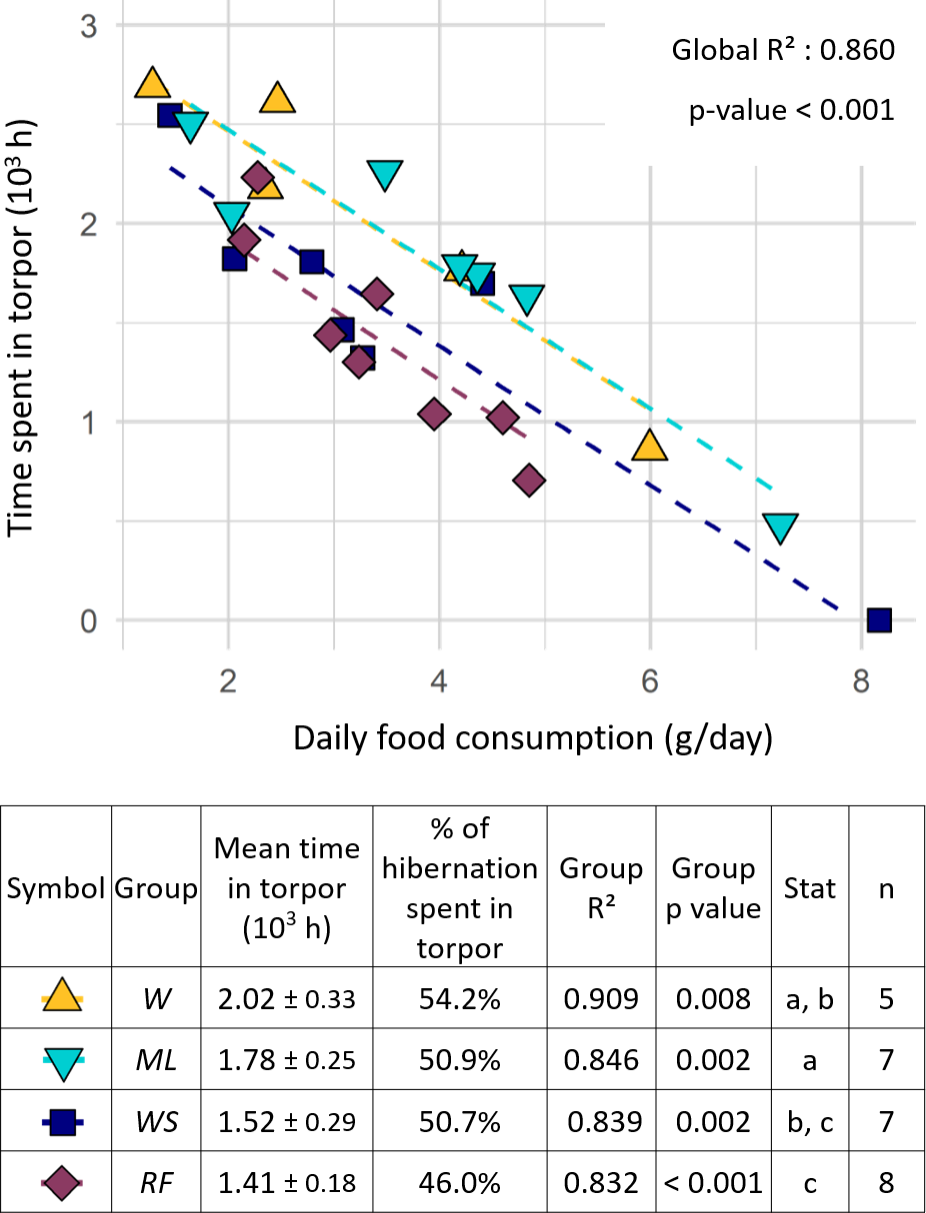
Total time spent in torpor by female hamsters (*n* = 27) during the hibernation period according to diet group and daily food consumption. The relationship between time spent in torpor and food consumption is indicated by linear regressions with group-specific intercepts. Group-specific regression *R*² and *p*-values are indicated in the table below the figure. Total torpor time is given as mean value ± s.e.m. for each group. The ‘Stat’ column indicates statistically significant differences between groups, where groups that do not share the same letter differ significantly from each other (Tukey, *p* < 0.04).

All hamsters survived hibernation. Except one female from the *WS* group that did not hibernate (thus excluded from figure 3’s middle point) all hamsters entered into torpor shortly after the beginning of the monitoring period (mean date: 23 October 2021 ± 1.7 days; 4 days after the start of monitoring). Start and end dates of hibernation (mean date: 14 March 2022 ± 3.9 days) did not differ between groups (lm, *p*_start_ = 0.451, *p*_end_ = 0.098). Nevertheless, at the individual level, a higher time spent in torpor induced a delayed end of hibernation (lm, *p* < 0.001). Diet by itself did not affect the time females spent in torpor (i.e. there was no difference between groups in one-way ANOVA, lm, *p* = 0.394). However, if the mass of consumed food was included in the model, torpor duration differed between groups (lm, *p* < 0.001; figure 4). *RF* and *WS* females spent less time in torpor than *W* and *ML* females. The difference was significant (Tukey, *p* < 0.04) except between *WS* and *W* (Tukey *W-WS, p* = 0.074). Model selection showed that the time spent in torpor was best predicted by hamster energy intake (lm, *p* > 0.001).

#### 3.1.3. Body mass dynamics during and after hibernation

All females lost body mass during hibernation and gained mass during post-hibernation (figure 3). Mean body mass was similar in all groups at the beginning (189.9 ± 2.5 g; lm, *p* = 0.902) and the end of hibernation (157.4 ± 2.9 g; lm, *p* = 0.780). The body mass variation through hibernation was best described by models containing either mass of consumed food (lm, *p* < 0.001) or energy intake (lm, *p* < 0.001). At the end of post-hibernation, body mass of females differed between groups (lm, *p* < 0.001). Females from the *RF* group had a significantly higher body mass (211.1 ± 5.8 g; Tukey *WS-RF*, *p* = 0.007, figure 3) than all other groups. Females body mass was similar and the lowest in the *W* and *ML* groups (171.9 ± 6.1 g; Tukey *W-ML*, *p* = 0.999). Body mass for *WS* females was intermediate (190.3 ± 6.0 g; Tukey *WS-W*, *p* = 0.018, *WS-ML*, *p* = 0.013). When relating female body mass at the end of winter with their nutritional intake, we found a significant positive effect for lipids (lm, *p* < 0.001, *R*² = 0.630), while such an effect was absent for protein, carbohydrate and overall energy intake (lm, *p*_energy_ = 0.309, *p*_protein_ = 0.217, *p*_carbo._ = 0.345).

### 3.2. Effects of winter on hamster reproduction

The hibernation end date had no effect on the date of first parturition (lm, *p* = 0.370), the number of litters (glm, *p* = 0.685) or the number of pups per female (glm, *p* = 0.846). To test for an effect of body mass on these parameters, independently of diet, the latter was included as a random effect in the following models. This showed that hamster body mass at the end of winter did not affect the date of first parturition (lmem, *p* = 0.723) or the number of pups produced (glmem, *p* = 0.307). Female body mass at the end of winter had a positive effect on the number of litters produced (glmem, *p* = 0.023). However, this effect was weak, as indicated by the low marginal coefficient of determination (*R*²*m* = 0.200).

### 3.3. Enclosure and reproduction

When released into the enclosures, hamsters from all groups quickly gained body mass. Female body mass averaged across the reproductive season was similar in all groups (297.5 ± 2.3 g; Tukey, *p* > 0.9), except for the *W* group, where it was significantly lower (263.0 ± 9.3 g; Tukey, *p* < 0.001). Some males (6 out of 12) succeeded to regularly pass from one sub-enclosure to another. This was also the case for one *RF* female that was thus excluded from reproduction analyses (see electronic supplementary material, table S4). Across all groups, a total of 320 pups were captured.

#### 3.3.1. Effects of diet on reproductive success

**Figure 5.**
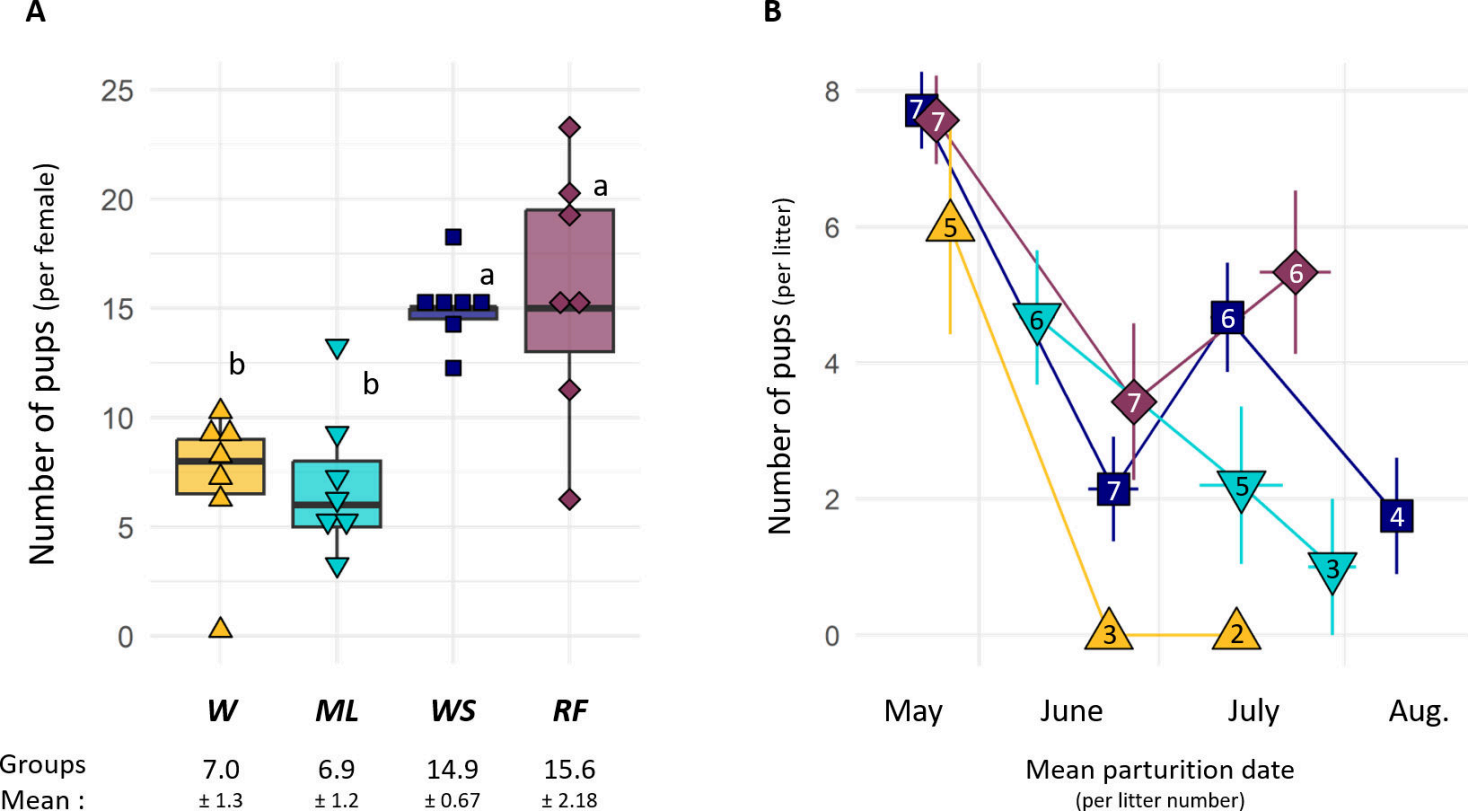
(A) Number of pups per female captured during the reproductive season (all litters combined) as a function of females’ diet. Letters indicate statistically significant differences between groups (Tukey, *p* < 0.001). The mean reproductive success (mean number of pups per female ± s.e.m.; *n* = 28) according to diet group is also indicated below. (B) Mean litter size (number of pups captured ± s.e.m.) and birth date ( ± s.e.m.) of litters, according to the litter number and hamsters’ group. Figures in points indicate how many females performed the corresponding litter (out of the 5 *W*, 6 *ML*, 7 *WS* and 7 *RF* females for which body temperature loggers were retrieved).

Diet had a significant effect on the reproductive output of females (glm, *p* < 0.001; figure 5A). *RF* and *WS* females on one hand, and *W* and *ML* females on the other hand produced a similar number of pups (Tukey *WS-RF*, *p* = 0.747; Tukey *W-ML*, *p* = 0.999). However, pups production was double in *RF* and *WS* groups than in *W* and *ML* ones (Tukey *W-WS, W-RF, WS-ML RF-ML*, *p* < 0.001; figure 5A). Only females from the *WS* group (four out of seven females) produced four litters, while females from the other groups produced between one and three litters (figure 5B). The total number of litters did not differ between groups (glm, *p* = 0.448). Nevertheless, the difference was significant (glm, *p* = 0.032) when only accounting for successful litters (i.e. at least one attributed pup). Successful litters were more numerous in the *RF* and *WS* groups (2.5 ± 0.3 and 3.0 ± 0.2 successful litters respectively, Tukey *RF-WS* = 0.172) than in the *ML* and *W* groups (1.7 ± 0.3 and 0.8 ± 0.2 successful litters respectively, Tukey *ML-W* = 0.107, Tukey *W-WS, W-RF, ML-WS, ML-RF* < 0.008).

For all groups, female’s first litter was significantly larger (mean size: 6.6 ± 0.5 pups; glm, *p* < 0.001; Tukey *1-2, 1-3, 1-4*, *p* < 0.003) than that of the second, third and fourth litter, which were similar (mean size: 2.3 ± 0.5, 3.7 ± 0.7 and 1.8 ± 0.9, respectively; Tukey *2-3, 3-4, 2-4*, *p* > 0.3). Litter size was diet dependent (glm, *p* < 0.001) and was higher in the *RF* group (5.3 ± 0.7) than in the *W* (2.9 ± 1.1, Tukey *RF-W*, *p* = 0.019) and *ML* groups (2.9 ± 0.7, Tukey *RF-ML*, *p* = 0.005). However, litter size in the *RF* group did not differ from that of the *WS* group (4.3 ± 0.6, Tukey *RF-WS*, *p* = 0.330; figure 5B). Litter size in the *WS*, *ML* and *W* group*s* did not differ significantly (Tukey *W-WS, ML-WS* and *ML-W*, *p* < 0.178). Furthermore, litter size was not correlated with the body mass of females before parturition (glm, *p* = 0.308). Interestingly, litter size was highly variable (0 to 10 observed pups per litter), and did not correlate with females’ reproductive success (*R*^2^ = 0.280, *p* = 0.166).

The mean date for first parturition was the 24 May 2022 ± 1.1 days in *WS*, *RF* and *W* females (Tukey *W-WS, W-RF, WS-RF*, *p* > 0.6). It was delayed by 16 ± 3 days in the *ML* group (lm, *p* < 0.001; Tukey *ML-W, ML-RF, ML-WS*, *p* < 0.001, figure 5B). The average delay between two litters was 30 ± 1 days, while the shortest delay was 19 days (observed in all groups). If females produced a large litter, parturition for the following litter was delayed (lm, *p* = 0.006). However, the delay between litters was not affected by female’s diet (lm, *p* = 0.207), their body mass before the previous parturition (lm, *p* = 0.264), or by litter number (lm, *p* = 0.101). The sex-ratio among trapped pups remained stable throughout the reproductive season, with around one third being females (♂/♀: 63.3/36.7 ± 0.03%; lm, *p* = 0.978).

#### 3.3.2. Effect of diet on pups’ growth

**Figure 6.**
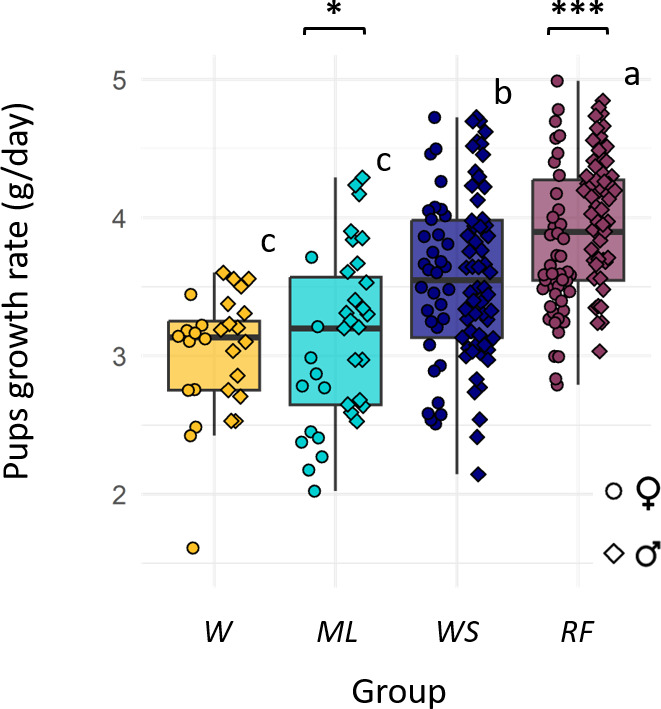
Pup growth rate (g day^−1^) as a function of their diet. For a better visualization, individual data points are separated horizontally according to sex (*n* = 249, females: dots to the left; males: diamonds to the right). Different colours correspond to the different diet groups. Asterisks and letters indicate statistically significant differences between sexes (Tukey, *p* < 0.013) and groups (Tukey, *p* < 0.001), respectively.

Pups that dispersed (*n* = 36 pups), pups whose birth date was unknown (the iButton of mothers were not recovered; *n* = 25 pups) and pups whose mother dispersed (*n* = 10 pups) were excluded from the growth analysis, leaving 249 pups for the latter. Pups’ body mass and age covaried linearly (*R*² = 0.671, *p* < 0.001). Diet strongly affected growth rates (lm, *p* < 0.001, figure 6). Pups’ growth rate were higher in *RF* (Tukey *RF-WS, p* < 0.001), intermediate in *WS* (Tukey *WS-ML*, *p* < 0.001) and similarly lower in *ML* and *W* (Tukey *ML-W*, *p* = 0.889). Sex also had an effect on growth rates but this was only significant in the *RF* (Tukey *RF*♂-♀, *p* = 0.001, figure 6) and the *ML* groups (Tukey *ML*♂-♀, *p* = 0.013), in which male pups gained an additional 0.355 and 0.672 g per day, respectively, when compared with female pups. Pups’ growth rate was also positively affected by the body mass of their mother (lmem, *p* < 0.001), while we found no litter size effect (lm, *p* = 0.077).

## 4. Discussion

Our study aimed at identifying mixes of crops that would be beneficial for hamster during the winter and the reproductive season. In accordance with our first prediction, torpor use was reduced in the most lipid-rich groups (*RF* and *WS;* figure 4). Diet did not consistently influence the hibernation end date, contradicting our second prediction (figure 3). During the post-hibernation period, hamsters gained body mass in all groups. In accordance with our third prediction, hamsters with a higher body mass at the end of winter produced more litters. Diet strongly affected reproductive success (figure 5). Reproductive failure was not observed in the *W* group (low-protein diet) contradicting our fourth prediction. In accordance with our fifth prediction, higher reproductive outputs were observed in the *WS* and *RF* groups, and these pups also grew faster, compared with the *W* and *ML* ones (figure 6).

### 4.1. Effects of diet during winter

During winter, hamsters showed a high variability of hibernation behaviours in the four groups, with a total time spent in torpor ranging from 0 to 2700 h (figure 4). Interestingly, hibernation traits covaried, with hamsters performing less torpor being the ones having consumed more food (and energy), and showing a lower body mass loss and an earlier end of hibernation. This means that hibernating hamsters spread along a behavioural continuum already described by Tissier *et al*. [20]. However, we did not observe any effect of the diet on behaviour or body mass changes during hibernation (figures 3 and 4). While the *WS* and *RF* groups ingested more lipids during hibernation than the *W* and *ML* groups, the latter ingested greater amounts of carbohydrates (figures 1C and 2 and electronic supplementary material, table S2). Given the variation in food consumption within groups, energy intake varied much more within than among groups (figure 1B). In addition, for all groups, the hibernation behaviour of hamsters depended mostly on their food/energy intake, so that hamsters with an increased food/energy intake had a reduced torpor expression. Thus, during hibernation, the greater lipid intake in the *WS* and *RF* groups alone did not translate into a significant reduction of torpor expression, or into an improved mass dynamic, as it had already been reported previously [30]. Such a high variability of behaviour within each group could be linked to non-nutritional factors, such as hereditary ones, as was recently demonstrated in garden dormice [39].

All groups showed a similar body mass loss over the 4.5 months of hibernation. However, differences between groups were detectible during the post-hibernation period, when a higher lipid intake in the *RF* and *WS* groups led to a greater mass gain (figure 3). One aspect in the decline of hamster populations has been linked with a reduced body condition at the start of the active season [11]. Hence, the planting of lipid-rich crops, such as provided to the *RF* and *WS* groups in our study, might be a promising way to improve hamster body condition at the end of winter.

### 4.2. Effect of winter on reproduction

How well hamsters make it through winter will have consequences for the upcoming reproductive season. A poor body condition will reduce the number of litters a female can produce and, hence, its reproductive output [20]. It might also delay the start of reproduction [15]. In our study, females with a higher body mass at the end of post-hibernation produced a larger number of litters. The number of litters produced per year has been identified as a critical parameter to achieve a positive population development in hamsters [9]. Hence, the higher body mass of females in the *RF* and *WS* groups at the end of post-hibernation is of relevance in this context (figure 3). However, in our study, diet in the *RF* and *WS* groups had a direct positive effect on reproductive success, so that it masked the much weaker body mass effect on reproductive success. Similarly, we did not find a relationship between the reproductive readiness of females (indicated by the date of their first litter) and their body mass at the end of winter. In our study, first litters occurred 35 days after the release in the *W*, *WS* and *RF* groups. Assuming a gestation period of 18 to 20 days, this suggests that successful mating occurred approximately 16 days after release into the enclosures [16]. This delay in the start of reproduction might indicate that females were not ready for reproduction at the time of release, or that the stress of release temporarily inhibited reproduction. However, first litters in the *ML* group were delayed by an additional 16 days (figure 5B), which might be a consequence of nutritional limitations in this group, as we will discuss below.

### 4.3. Effects of diet on reproduction

During the active season, all females attempted reproduction and produced at least one litter (figure 5). By contrast to what had been reported by Tissier *et al.* [10,11] in laboratory and semi-natural conditions, the females fed with the low-protein *W* diet produced a mean of seven pups each. That could be explained by females’ access to a higher number of males (three versus one, leading to a real choice of the better male) and larger enclosures (1900 versus 32 m²) with greater biodiversity. Indeed, additionally to the sown crops, weeds (mainly goosefoot *Chenopodium* sp. and mayweed *Matricaria* sp.) made up a significant part of the plant cover inside the enclosures (20% or more) that hamsters could eat. As an omnivorous species, hamsters have been observed (via camera traps) consuming supplemented crop grains, fresh plant parts, arthropods and even voles (*Microtus* sp.), of which dry mass protein content range from 20% to 60% [31,40]. Therefore, they might have preserved the *W* group from protein deficiencies, and enabled the production of their first litter. Similarly, the *ML* females’ reproductive success (seven pups per female) suggests that *ML* females have found an external supply of niacin or its precursor tryptophan either through the consumption of lablab beans, the green parts of plants, or other food sources found in their enclosures. Nevertheless, the similar reproductive outputs in *W* and *ML* groups suggest that *ML* females did not really benefit from the higher protein content of the lablab beans. This might be due to a low palatability of lablab beans [41]. Furthermore, the onset of reproduction was delayed in *ML* females, which may be due to a certain protein deficiency as observed in greater long-tailed hamster [42].

In comparison with the *W* and *ML* groups, reproductive outputs were considerably larger in the *RF* and *WS* groups*,* in which females produced a mean of 15 pups each. This was notably permitted by both more numerous and larger successful litters in those two groups. *WS* and *RF* females consistently produced approximately three successful litters. Such number of litters is considerably higher than what is currently observed in wild populations (less than one litter per year) and consistent with a positive population demography [9,26]. Nevertheless, a low reproductive success in the wild might also be linked to disturbance (harvest, ploughing, etc.), which did not occur in the enclosure [43]. Interestingly, during reproduction, the mean delay between successive litters was 30 days, and could even be reduced to 19 days (observed for seven females). Considering minimal length of 18 days for gestation and 20 days for rearing, this means that females were often pregnant and lactating at the same time, confirming previous data [16]. Because of the high energy requirements associated with gestation and lactation, this emphasizes the importance of efficient nutrient and energy acquisition [44]. The higher availability of (structural) proteins and (energy-rich) lipids in the *RF* and *WS* diets thus benefit reproduction and explain why pups in these groups showed an accelerated growth, improving their nutritional intake during both suckling and autonomous feeding. Such rapid growth might also be highly beneficial as it could enhance winter survival (as shown in Columbian ground squirrel; [45]), and allow first-born pups to reproduce before their first hibernation [46].

### 4.4. Agronomical implications

Our results show the importance of diversifying hamsters’ environment to provide lipids and proteins. Proteins are contained in seeds and green parts of legumes crops [40]. Alternatively, farmland biodiversity (weeds, invertebrates and/or micromammals) offers protein supplements, encouraging for more environmentally friendly agricultural practices. By contrast, lipid sources are scarce in the environment, and mostly found in oleaginous seeds (e.g. rapeseed and soybean used in this study; [40]). Moreover, in the wild, crop graining phenology and timing of the agricultural harvest are also key factors to consider for conservation measures [43]. Future studies might aim at (i) matching legume and oleaginous crops phenology with hamster food consumption during both reproduction and hoarding behaviour. This would also (ii) help to promote vegetal cover during the active season and protect hamsters from predation. In light of this, crop associations of complementary phenology, such as winter wheat with spring soybean, or winter rapeseed with spring fava bean (corresponding to the *WS* and *RF* associations tested in this study), appear beneficial to achieve these goals. Additionally, these associations were shown to be agronomically viable and were already implemented by some Alsatian farmers, using relay or co-cropping techniques. Their benefits as agro-environmental practices could extend beyond the hamster conservation, and promote farmland biodiversity as a whole.

## Data Availability

All data, code and materials used in this study are available online in the Environment and Society Data Inventory (InDoRES) data repository [47]. Supplementary material is available online [48].
